# Possible misdiagnosis, inappropriate empiric treatment, and opportunities for increased diagnostic testing for patients with vulvovaginal candidiasis—United States, 2018

**DOI:** 10.1371/journal.pone.0267866

**Published:** 2022-04-28

**Authors:** Kaitlin Benedict, Meghan Lyman, Brendan R. Jackson

**Affiliations:** Mycotic Diseases Branch, Centers for Disease Control and Prevention, Atlanta, Georgia, United States of America; University of Palermo: Universita degli Studi di Palermo, ITALY

## Abstract

Vulvovaginal candidiasis (VVC) is a common cause of vaginitis, but the national burden is unknown, and clinical diagnosis without diagnostic testing is often inaccurate. We aimed to calculate rates and evaluate diagnosis and treatment practices of VVC and recurrent vulvovaginal candidiasis (RVVC) in the United States. We used the 2018 IBM® MarketScan® Research Databases, which include health insurance claims data on outpatient visits and prescriptions for >28 million people. We used diagnosis and procedure codes to examine underlying conditions, vaginitis-related symptoms and conditions, diagnostic testing, and antibacterial and antifungal treatment among female patients with VVC. Among 12.3 million female patients in MarketScan, 149,934 (1.2%) had a diagnosis code for VVC; of those, 3.4% had RVVC. The VVC rate was highest in the South census region (14.3 per 1,000 female patients) and lowest in the West (9.9 per 1000). Over 60% of patients with VVC did not have codes for any diagnostic testing, and microscopy was the most common test type performed in 29.5%. Higher rates of diagnostic testing occurred among patients who visited an OB/GYN (53.4%) compared with a family practice or internal medicine provider (24.2%) or other healthcare provider types (31.9%); diagnostic testing rates were lowest in the South (34.0%) and highest in the Midwest (41.0%). Treatments on or in the 7 days after diagnosis included systemic fluconazole (70.0%), topical antifungal medications (19.4%), and systemic antibacterial medications (17.2%). The low frequencies of diagnostic testing for VVC and high rates of antifungal and antibacterial use suggest substantial empiric treatment, including likely overprescribing of antifungal medications and potentially unnecessary antibacterial medications. These findings support a need for improved clinical care for VVC to improve both patient outcomes and antimicrobial stewardship, particularly in the South and among non-OB/GYN providers.

## Introduction

Vaginitis is a common complaint in the outpatient setting [1]. *Candida* spp. cause approximately 30% of vaginitis episodes, with an estimated 75% of women affected by vulvovaginal candidiasis (VVC) during their lifetimes [1–3]. Recurrent VVC (RVVC), generally defined as ≥3 VVC episodes per year, affects approximately 5–10% of women and is more frequently associated with non-*albicans Candida* species and antifungal resistance [3–5]. The true prevalence of VVC and RVVC are difficult to estimate because of the absence of public health surveillance for these conditions, the nonspecific clinical presentation, and insufficient use of diagnostic testing resulting in incorrect diagnosis [3].

Diagnosis of VVC is challenging because the symptoms (e.g., vaginal discharge, pruritus, pain, swelling, and redness) can resemble other common causes of vaginitis such as bacterial vaginosis or trichomoniasis. Therefore, diagnosis of VVC based on signs and symptoms alone is unreliable and can be improved by point-of-care testing (i.e., wet mount microscopy, potassium hydroxide [KOH] test to assess for amine odor, or vaginal pH test) or laboratory-based testing (culture, PCR). CDC guidance recommends that wet mount microscopy be performed for all women with signs and symptoms of VVC and that wet mount should be followed by culture if the results are negative [6]. The guidelines further recommend culture and PCR for patients with severe VVC or recurrent episodes. Despite the low cost and convenience of point-of-care testing for VVC, some studies show that these tests do not appear to be commonly performed, leading to inappropriate empiric treatment, prolonged symptoms, and repeat healthcare visits [2]. VVC can usually be successfully treated using short courses of topical or oral azole antifungals, although severe VVC, non-*albicans* VVC, or RVVC often require a longer treatment course [6, 7].

Rates and diagnosis and treatment practices of VVC and RVVC in the United States have not been well described on a national level. Therefore, we aimed to describe these patterns using a large commercial health insurance dataset, with the goal of informing clinical practice.

## Methods

We used data from the 2018 IBM® MarketScan® Commercial and the Medicare Supplemental databases, which include health insurance claims data from outpatient visits, outpatient prescriptions, and hospitalizations for >28 million employees, dependents, and retirees across the United States.

To query the data, we used Treatment Pathways, a web-based platform with data from people whose health insurance plans contribute prescription drug information to the MarketScan databases (94% of enrollees). We established three non-mutually exclusive cohorts of female patients. First, we identified those with an International Classification of Diseases, Tenth Revision, Clinical Modification (ICD-10-CM) diagnosis code B37.3 for VVC at an outpatient visit in 2018. The index date was the first date this code was used in 2018, and we limited the analysis to patients with continuous insurance enrollment in the 30 days before and the 30 days after the index date. Among females with VVC, we established another cohort of patients with continuous insurance enrollment in the 30 days before and the 365 days after the VVC index date and identified those with RVVC, defined as ≥3 episodes of VVC within one year, with each episode separated by ≥30 days. Lastly, as a comparison cohort, we identified female patients with an outpatient visit for a routine gynecological examination (ICD-10-CM codes Z01.411 or Z01.419) in 2018 and who were continuously enrolled in the 30 days before and the 30 days after the date either of those codes were first used (the index date).

For each cohort, we described demographic characteristics and other features of interest using ICD-10-CM codes and Current Procedural Terminology (CPT) codes (S1 Table). This included underlying conditions (on or in the 30 days before the index date) and medications (in the 30 days before the index date) commonly associated with increased risk for VVC, VVC and other vaginal-related symptoms and conditions and diagnostic testing (on or in the 30 days before and in the 30 days after the index date), and antibacterial and antifungal treatment (on or in the 7 days after the index date). For the RVVC cohort, these features relate to the third VVC episode. We also calculated VVC rates per 1,000 female patients in MarketScan to assess regional differences. MarketScan data are fully de-identified, so the analysis was not subject to review by the Centers for Disease Control and Prevention institutional review board.

## Results

Among the 12.3 million female patients with any healthcare encounter in 2018, 149,934 had a diagnosis code for VVC at an outpatient visit. The VVC rate per 1,000 female patients in MarketScan was highest in the South Census region (14.3) compared with the Northeast (11.5), Midwest (10.4), and West (9.9). The median age of patients with VVC was 34 years (range 0–102), and nearly two-thirds were aged 18–44 years (Table 1). Of the 109,667 patients with VVC who were continuously enrolled in the year after the index date, 3,689 (3.4%) had RVVC. The comparison cohort of all females with a routine gynecologic exam consisted of 2,743,624 patients.

**Table 1:** Demographic features, underlying conditions, and pre-diagnosis medications among patients with vulvovaginal candidiasis, recurrent vulvovaginal candidiasis, and females with an outpatient gynecologic visit.

	VVC		RVVC		Females with an outpatient gynecologic visit in 2018	
	n = 149,934	%	n = 3,689	%	n = 2,743,624	%
Median age, years (range)	34	(0–102)	34	(1–95)	42	(0–98)
Age group, years						
0–17	9,297	6.2%	69	1.9%	26,017	0.9%
18–34	66,250	44.2%	1,853	50.2%	860,892	31.4%
35–44	30,946	20.6%	797	21.6%	625,190	22.8%
45–54	24,654	16.4%	577	15.6%	658,572	24.0%
55–64	15,546	10.4%	320	8.7%	532,663	19.4%
≥65	3,241	2.2%	73	2.0%	40,200	1.5%
U.S. Census region of primary beneficiary’s residence						
Northeast	25,231	16.8%	727	19.7%	589,363	21.5%
Midwest	26,987	18.0%	639	17.3%	507,676	18.5%
South	77,595	51.8%	1,952	52.9%	1,322,200	48.2%
West	19,814	13.2%	363	9.8%	321,807	11.7%
Missing	307	0.2%	8	0.2%	2,578	0.1%
Conditions on or in the 30 days before the index date						
Diabetes	9,979	6.7%	394	10.7%	63,980	2.3%
Pregnancy	2,794	1.9%	44	1.2%	9,779	0.4%
Hematologic malignancy	171	0.1%	4	0.1%	2,809	0.1%
HIV	102	0.1%	8	0.2%	1,001	0.1%
Transplant	78	0.1%	2	0.1%	1,579	0.0%
Medications prescribed in the 30 days before the index date						
Systemic antibacterial medications	25,738	17.2%	561	15.2%	195,447	7.1%
Aminoglycoside	19	0.1%	1	0.2%	178	0.1%
Beta-lactam	9	0.0%	0	0.0%	29	0.0%
Cephalosporin	4,595	17.9%	80	14.3%	26,524	13.6%
Chloramphenicol & combination	0	0.0%	0	0.0%	0	0.0%
Clindamycin	1,077	4.2%	32	5.7%	7,638	3.9%
Erythromycin/macrolide	3,815	14.8%	118	21.0%	42,845	21.9%
Penicillins	10,441	40.6%	173	30.8%	65,509	33.5%
Tetracyclines	2,884	11.2%	84	15.0%	27,349	14.0%
Sulfonamide & combination	2,829	11.0%	64	11.4%	18,265	9.3%
Urinary anti-infectives	3,434	13.3%	89	15.9%	21,756	11.1%
Metronidazole	3,894	2.6%	155	4.2%	10,720	0.4%
Steroids	6,654	4.4%	179	4.9%	83,334	3.0%
Estrogen-containing contraceptives	15,992	10.7%	480	13.0%	268,512	9.8%
Hormone replacement therapy	2,734	1.8%	90	2.4%	50,912	1.9%
Progestin-only contraceptives	1,479	1.0%	45	1.2%	27,251	1.0%

The most common underlying condition we evaluated was diabetes mellitus, in 6.7% of patients with VVC, 10.7% of patients with RVVC, and 2.3% of patients with a routine gynecologic exam. Pregnancy diagnosis codes were listed for 1.9% of patients with VVC, 1.2% of patients with RVVC, and 0.4% of patients with a routine gynecologic exam. Systemic antibacterial medications were prescribed in the 30 days before the index date for 17.2% of patients with VVC, 15.2% of patients with RVVC, and 7.1% of patients with a routine gynecologic exam.

On the index date, 35.0% of patients with VVC and 39.3% of patients with RVVC visited an obstetrician-gynecologist (OB/GYN), and 28.3% of patients with VVC and 26.6% of patients with RVVC visited a family practice provider or internal medicine provider. For patients with VVC, other diagnosis codes commonly assigned on or before the index date included routine gynecologic exam (16.9%), acute vaginitis or vulvitis (15.7%), screening for sexually transmitted infection (12.3%), and urinary tract infection or acute cystitis (10.8%) (Table 2). Diagnosis codes for a routine gynecologic exam were less frequently listed on or before the index date for patients with RVVC (8.6%).

**Table 2:** Vulvovaginal candidiasis-related symptoms and conditions.

	On or in the 30 days before the index date				In the 30 days after the index date			
	VVC		RVVC		VVC		RVVC	
	n = 149,934	%	n = 3,689	%	n = 149,934	%	n = 3,689	%
Urinary tract infection or acute cystitis	16,244	10.8%	362	9.8%	5,247	3.5%	136	3.7%
Leukorrhea	17,488	11.7%	542	14.7%	4,151	2.8%	175	4.7%
Dysuria	13,351	8.9%	260	7.0%	3,296	2.2%	97	2.6%
Pruritus vulvae	1,308	0.9%	48	1.3%	376	0.3%	18	0.5%
Urinary frequency	3,933	2.6%	92	2.5%	1,312	0.9%	47	1.3%
Vulvodynia	218	0.1%	25	0.7%	121	0.1%	9	0.2%
Contact with and suspected exposure to sexually transmitted infection	2,698	1.8%	83	2.2%	738	0.5%	31	0.8%
Routine gynecological examination	25,375	16.9%	319	8.6%	5,710	3.8%	144	3.9%
Acute vaginitis or vulvitis	23,504	15.7%	800	21.7%	6,273	4.2%	247	6.7%
Screening for sexually transmitted infection	18,480	12.3%	464	12.6%	3,992	2.7%	164	4.4%
Screening for other infections	2,295	1.5%	68	1.8%	521	0.3%	8	0.2%
Trichomoniasis	672	0.4%	22	0.6%	309	0.2%	8	0.2%
Gonorrhea	198	0.1%	5	0.1%	162	0.1%	3	0.1%
Chlamydia	273	0.2%	12	0.3%	132	0.1%	3	0.1%

Overall, 37.1% of patients with VVC and 43.8% of patients with RVVC received codes for diagnostic testing for VVC. Higher rates of diagnostic testing occurred among patients who visited an OB/GYN on the index date (53.4% for patients with VVC and 60.4% for patients with RVVC) compared with a family practice or internal medicine provider (24.2% for patients with VVC and 28.1% for patients with RVVC) or other healthcare provider types (31.9% for patients with VVC and 37.3% for patients with RVVC) (Table 3). Microscopy was the most common test type performed, in 29.5% of patients with VVC and 31.5% of patients with RVVC. Antifungal susceptibility testing was performed for 5.2% of patients with VVC and 4.9% of patients with RVVC. By region, the proportion of patients with VVC who had any diagnostic testing performed was lowest in the South (34.0%) and highest in the Midwest (41.0%), and the proportion of patients with RVVC who had any diagnostic testing performed was lowest in the South (39.1%) and highest in the Northeast (52.3%).

**Table 3:** Diagnostic testing by healthcare provider type visited on the index date, among patients with vulvovaginal candidiasis.

	Obstetrics/gynecology		Family practice or internal medicine		Other	
** VVC**	**n = 52,523**	**%**	**n = 42,503**	**%**	**n = 55,956**	**%**
Vaginal pH test	738	1.4%	140	0.3%	560	1.0%
Microscopy	19,532	37.2%	7,061	16.6%	12,585	22.5%
Fungal culture	1,021	1.9%	350	0.8%	920	1.6%
*Candida* nucleic acid test	10,234	19.5%	3,585	8.4%	5,813	10.4%
Any of the above	28,046	53.4%	10,301	24.2%	17,857	31.9%
Antifungal susceptibility test	2,319	4.4%	2,456	5.8%	3,148	5.6%
**RVVC**	**n = 1,448**	**%**	**n = 982**	**%**	**n = 1,276**	**%**
Vaginal pH test	34	2.3%	3	0.3%	28	2.2%
Microscopy	619	42.7%	199	20.3%	352	27.6%
Fungal culture	107	7.4%	19	1.9%	41	3.2%
*Candida* nucleic acid test	301	20.8%	93	9.5%	151	11.8%
Any of the above	874	60.4%	276	28.1%	476	37.3%
Antifungal susceptibility test	51	3.5%	57	5.8%	74	5.8%

Most patients (70.0% of those with VVC and 65.8% of those with RVVC) were prescribed systemic fluconazole on or in the 7 days after the index date (Table 4). Patients with RVVC more frequently received >2 doses of fluconazole (29.0%) compared with all patients with VVC (19.3%). Topical antifungal medications were prescribed for 19.4% of patients with VVC and 27.3% of patients with RVVC, most commonly terconazole. Systemic antibacterial medications were prescribed for 16.8% of patients with VVC and 15.7% of patients with RVVC. Specifically, antibacterial medications were prescribed for 44.3% of the patients with VVC who also received a diagnosis code for urinary tract infection or acute cystitis and for 11.9% who also received a diagnosis code for acute vaginitis or vulvitis. Among patients with VVC who visited an OB/GYN on the index date, 8.4% were prescribed systemic antibacterial medications, compared with 20.6% who visited a family practice or internal medicine provider and 21.6% who visited another type of healthcare provider.

**Table 4:** Antibacterial and antifungal treatment among patients with vulvovaginal candidiasis.

	VVC		RVVC	
Medications on or in the 7 days after index date	n = 149,934	%	n = 3,689	%
Systemic antifungal medication	105,100	70.1%	2,447	66.3%
Fluconazole	104,877	70.0%	2,428	65.8%
One dose	37,610	35.9%	653	26.9%
Two doses	47,105	44.9%	1,078	44.4%
More than two doses	20,248	19.3%	704	29.0%
Topical antifungal medication	29,018	19.4%	662	27.3%
Clotrimazole	6,249	21.5%	116	17.5%
Miconazole	109	0.4%	3	0.5%
Terconazole	12,840	44.2%	402	60.7%
Butoconazole	364	1.3%	14	2.1%
Nystatin	10,273	35.4%	166	25.1%
Ketoconazole	692	2.4%	18	2.7%
Systemic and topical antifungal medication	14,388	9.6%	399	10.8%
Systemic or topical antifungal medication	119,730	79.9%	2,773	75.2%
Systemic antibacterial medication*	25,115	16.8%	580	15.7%
Metronidazole	11,299	7.5%	314	8.5%

*excludes metronidazole

## Discussion

VVC was a common condition in this study of patients with private health insurance, affecting 1.2% of females in 2018, based on ICD-10-CM coding. Among those patients, 3.5% had ≥3 VVC episodes within one year, indicating that these infections can cause prolonged morbidity. We observed low frequencies of diagnostic testing for VVC, with many patients subsequently receiving apparent empiric antifungal treatment. Some patients with VVC also received systemic antibacterial treatment, perhaps further reflecting empiric treatment for presumed simultaneous bacterial vaginosis or urinary tract infections.

Previous estimates that 75% of women experience at least one episode of VVC in their lifetime and that 5–10% will experience RVVC are not well-documented. Recent information about the incidence of VVC and RVVC is also somewhat lacking, though a few studies have calculated the incidence of self-reported healthcare-provider diagnosed VVC to be approximately 5% among reproductive-age women [5, 8, 9]. The lower incidence of VVC and RVVC observed in this study could be partly due to our use of a broader age range or due to administrative codes, which are likely less sensitive than self-report data; the sizeable proportion of patients with VVC who had concurrent diagnosis codes for acute vaginitis or vulvitis also supports this hypothesis. Underscoring the lack of sensitivity of the VVC code, 23% of 403,206 women in the database with an acute vaginitis or vulvitis code but not a concurrent VVC code received systemic antifungal treatment. Furthermore, the true burden of VVC is undoubtedly higher, as patients self-diagnose and self-treat with over-the-counter antifungal medication. Although MarketScan data slightly over-represent the South compared with the general population, the regional variations in VVC rates we observed suggest variability in provider-diagnosed VVC. Whether this reflects differences in susceptibility to VVC and disease burden, care-seeking behavior, or diagnosis warrants further study. Previous studies suggest that the South may have a higher burden of VVC [5] and higher rates of outpatient fluconazole prescribing [10], bacterial sexually transmitted infections [11], and antibacterial medication prescribing [12], which could lead to greater risk for developing VVC.

Consistent with previous studies, clinical risk factors for VVC, such as systemic antibacterial medication use, hormonal contraceptive use, and diabetes, were more frequent among patients with VVC than among patients with a routine gynecologic exam in this analysis. In particular, we found that pre-diagnosis use of systemic antibacterial medication was more than twice as common among patients with VVC than patients with a routine gynecologic exam. Antibacterial use, presumably for infections unrelated to VVC, is widely known to trigger the development of VVC by disrupting the vaginal microbiome, allowing for *Candida* overgrowth [13]. Because many other studies have clearly shown that antibacterial use and other factors can predispose females to developing VVC, we chose not to perform a case-control analysis to further confirm these well-established associations. Notably, many patients in this study did not have any apparent predisposing factors, supporting literature showing that VVC is a common infection among otherwise healthy females [13, 14]. However, our analysis likely underestimates the proportions of patients with specific predisposing factors if no ICD-10 code was used in the 30 days before the index date. The similarities we observed between all patients with VVC and those with RVVC in terms of demographic features, underlying conditions (with the exception of diabetes), and pre-index date medications are consistent with the hypothesis that other factors not accounted for in this analysis, such as genetic susceptibility, likely contribute to increased risk for recurrent episodes of VVC [15].

Over 60% of all patients with VVC and over 50% of patients with RVVC did not have codes for any diagnostic test, suggesting incomplete coding practices, extensive empiric treatment, or both. This finding is concerning given that VVC diagnosis based on clinical findings alone is insufficient [15]. Previous studies have found that misdiagnosis of VVC is high (>75%), regardless of diagnostic techniques used [15]. Our finding that all types of diagnostic testing were more common among patients who visited OB/GYNs may indicate greater ease of testing or greater awareness of the importance of testing among OB/GYNs compared with other provider types. In particular, there was poor adherence to testing practices recommended in CDC guidelines for RVVC (PCR and culture), likely limiting the ability to detect non-*albicans* species. However, our analysis did not capture tests performed earlier than 30 days before or later than 30 days after patients’ third VVC episodes. Similarly, antifungal susceptibility testing, which is sometimes recommended for RVVC, was very uncommon (≤5%) and did not vary substantially by provider type or recurrence of VVC. Lastly, the lower testing rates in the South calls into question whether the increased incidence of documented VVC reflects true illness burden and suggests that at least some of the difference could be driven by greater empiric diagnosis and possible misdiagnosis as other conditions.

The considerable proportion of patients with concurrent diagnosis codes for other infections with symptoms similar to VVC such as bacterial vaginosis (which does not have a specific ICD-10-CM code and is typically coded as “acute vaginitis or vulvitis”), sexually transmitted infections, and urinary tract infections also supports our finding of low diagnostic testing for VVC. Coupled with substantial rates of antibacterial treatment (17%), these results may point to empiric treatment for multiple conditions. A portion of the antibacterial treatment could be explained by ongoing symptoms or recurrence of a previous urinary tract infection. However, antibiotic treatment among patients with “acute vaginitis or vulvitis” diagnosis codes, particularly the high metronidazole use despite low frequency of trichomoniasis, suggests that some patients with VVC diagnosis codes were also being treated for presumed bacterial vaginosis. An alternative explanation involves infections of mixed etiologies, although this is relatively uncommon (<6%) in other epidemiologic studies of vaginitis [2, 16].

Over two-thirds of patients received systemic fluconazole as treatment, consistent with previous reports [14]. This high proportion is notable because CDC treatment guidelines also suggest a range of intravaginal treatment options (used in only 20% of patients in our analysis) for non-severe VVC. We were unable to assess VVC severity, but severe VVC, which warrants systemic therapy, is typically substantially less common than milder forms. Strangely, a higher proportion of patients with RVVC received topical antifungal prescriptions compared with all patients with VVC, whereas CDC treatment guidelines recommend oral fluconazole therapy for most RVVC cases [6]. However, it is not surprising that longer oral fluconazole treatment courses were more common in patients with RVVC. A relatively small proportion (10%) of patients with VVC received both systemic and topical antifungals, which could reflect either a lack of improvement with initial treatment or excessive treatment. Approximately 20% of patients with VVC and 25% of patients with RVVC were not treated with prescription antifungals, but the dataset does not include information about over-the-counter antifungal treatments, which are widely available. Altogether, the high rates of prescription antifungal use we observed, along with low rates of diagnostic testing, suggest extensive empiric treatment. Continuing to understand treatment practices for VVC will be important given the approval in mid-2021 of ibrexafungerp, which represents a new antifungal class and is reported to be substantially more expensive than fluconazole [17].

Because health insurance claims data are not specifically designed for public health surveillance or research, our results are subject to inherent limitations related to validity of administrative codes for detecting infections, clinical features, and diagnostic tests. However, these data offer the advantage of a large sample size, which can be difficult to achieve with other study designs and data sources. MarketScan data are broadly representative of the segment of the US population with private health insurance, though they do not necessarily represent persons with other types of health insurance or those without health insurance.

In summary, VVC was a commonly coded condition among female patients with private health insurance, with higher rates in the South compared with other regions of the United States. Few patients underwent diagnostic testing for VVC (which is even more concerning for those with RVVC), suggesting possible misdiagnosis and overprescribing of antifungals, which could contribute to antifungal resistance on a broader scale. A sizeable proportion of patients with VVC received simultaneous prescriptions for systemic antibacterial medications, many of which were likely for bacterial vaginosis and urinary tract infections, further raising questions about excessive empiric therapy. These findings support those from other studies suggesting a need for improved clinical care for VVC to improve antifungal stewardship and patient outcomes.

## Supporting information

S1 Table International Classification of Diseases, Tenth Revision, Clinical Modification (ICD-10) and Current Procedural Terminology (CPT) codes used to define features of interest. (DOCX)
