# Supplementary material for: Possible misdiagnosis, inappropriate empiric treatment, and opportunities for increased diagnostic testing for patients with vulvovaginal candidiasis—United States, 2018
Source: PLoS One. 2022 Apr 28;17(4):e0267866. doi: 10.1371/journal.pone.0267866 (PMC9049332; doi:10.1371/journal.pone.0267866)
Supplement: S1 Table — (DOCX) [file pone.0267866.s001.docx]

**S1 Table. International Classification of Diseases, Tenth Revision, Clinical Modification (ICD-10) and Current Procedural Terminology (CPT) codes used to define features of interest.**

| **Description** | **Definition** |
| --- | --- |
| Vulvovaginal candidiasis | ICD-10 code B37.3 |
| Diabetes | ICD-10 codes E08-E13 |
| Hematologic malignancy | ICD-10 codes C81-C96 |
| Solid organ or stem cell transplant | ICD-10 codes T86, Z94 |
| HIV | ICD-10 code B20 |
| Pregnancy | ICD-10 codes Z33.1, Z34, O09 |
| Urinary tract infection or acute cystitis | ICD-10 codes N30.00, N30.01, N39.0 |
| Leukhorrhea | ICD-10 code N89.8 |
| Dysuria | ICD-10 codes R30.0, R30.9 |
| Pruritus vulvae | ICD-10 code L29.2 |
| Urinary frequency | ICD-10 code R35.0 |
| Vulvodynia | ICD-10 codes N94.818, N94.819 |
| Contact with and suspected exposure to sexually transmitted infection | ICD-10 code Z20.2 |
| Routine gynecological examination | ICD-10 codes Z01.411, Z01.419 |
| Acute vaginitis or vulvitis | ICD-10 codes N76.0, N76.2, N76.81, N76.89 |
| Screening for sexually transmitted infection | ICD-10 code Z11.3 |
| Screening for other infections | ICD-10 code Z11.8, Z11.9 |
| Trichomoniasis | ICD-10 code A59 |
| Gonorrhea | ICD-10 code A54 |
| Chlamydia | ICD-10 code A56 |
| Vaginal pH test | CPT code 82120 |
| Microscopy | CPT code 87220, 87205, 87206, 87210 |
| Fungal culture | CPT codes 87101, 87102, 87106, 87107 |
| *Candida* nucleic acid test | CPT codes 87480, 87481, 87482 |
| Antifungal susceptibility test | CPT code 87186 |
